# Tuberculosis and Genius

**Published:** 1914-12

**Authors:** 


**Tuberculosis and Genius,** Arthur C. Jacobson, Brooklyn, Ther. Gaz., Aug. 1914. The author mentions Robert Louis Stevenson, Chopin, Keats, Marie Bashkirtseff, Schiller, John Milton, John Locke, Alexander Pope, Percy Bysshe Shelley, Tom Hood, Laurence Sterne, Thomas De Quincey, Elizabeth Barrett Browning, Mohere, Henry Thoreau, Goethe, Balzac, Jane Austen, Samuel Butler, Edward Gibbon, Voltaire, Francis Beaumont, Walter Scott, Dr. Johnson, Baruch Spinoza, Georges de Guérin, David Gray, Amiel, Washington Irving, John R. Green, Richard Baxter, Charlotte Bronte and her almost equally distinguished sisters, Emily and Ann, Rousseau, John Ruskin, Charles Kingsley, Robert Southey, Nathaniel Hawthorne, Toru Dutt, Robert Pollok, Hannah More, Pierre Jean de Béranger, William Ellery Channing, Immanuel Kant, ''Thomas Ingoldsby'' (Richard Harris Barham) and James Ryder Randall. In addition to these may be mentioned Mozart, Descartes, Mérimée, Richelieu, Raphael, Bastien-Lepage, Jacquemart, Trutat, Watteau, Paganini, von Weber, Nevin, Purcell, Rachel, Laennec, Bichat, Cardinal Manning, Rush, Trudeau, Godman, William Pepper (2nd), Calvin, Cicero, Cecil Rhodes, Saint Francis of Assisi and the great surgeon Dupuytren, and concludes with a biograph of Francis Thompson, English poet and medical student.

